# Coverage Intervals

**DOI:** 10.6028/jres.126.004

**Published:** 2021-03-03

**Authors:** Sara Stoudt, Adam Pintar, Antonio Possolo

**Affiliations:** 1 Smith College, Northampton, MA 01063, USA; 2 National Institute of Standards and Technology, Gaithersburg, MD 20899, USA

**Keywords:** Bayesian model, coverage interval, Hodges-Lehmann, median, Monte Carlo method, non-parametric, prediction interval, predictive interval, reference material, tolerance interval, Type A evaluation, Weibull distribution

## Abstract

Since coverage intervals are widely used expressions of measurement uncertainty, this contribution reviews coverage intervals as defined in the *Guide to the Expression of Uncertainty in Measurement* (GUM), and compares them against the principal types of probabilistic intervals that are commonly used in applied statistics and in measurement science.

Although formally identical to conventional confidence intervals for means, the GUM interprets coverage intervals more as if they were Bayesian credible intervals, or tolerance intervals.

We focus, in particular, on a common misunderstanding about the intervals derived from the results of the Monte Carlo method of the GUM Supplement 1 (GUM-S1), and offer a novel interpretation for these intervals that we believe will foster realistic expectations about what they can deliver, and how and when they can be useful in practice.

## 1 Introduction

The *Guide to the Expression of Uncertainty in Measurement* (GUM) [1–3] states:

“In many industrial and commercial applications, as well as in the areas of health and safety, it is often necessary to provide an interval about the measurement result that may be expected to encompass a large fraction of the distribution of values that could reasonably be attributed to the quantity subject to measurement. Thus the ideal method for evaluating and expressing uncertainty in measurement should be capable of readily providing such an interval, in particular, one with a coverage probability or level of confidence that corresponds in a realistic way with that required” — GUM 0.4

The intervals the GUM contemplates are of the form *y* ±*U* (*y*), where *y* denotes an estimate of the true value of the measurand, and *U* (*y*) denotes an expanded uncertainty associated with *y*, for some specified level of confidence. The GUM calls such intervals *coverage intervals* for the following reason:

“The terms confidence interval (C.2.27, C.2.28) and confidence level (C) have specific definitions in statistics and are only applicable to the interval defined by *U* when certain conditions are met, including that all components of uncertainty that contribute to *u*_c_(*y*) be obtained from Type A evaluations. Thus, in this Guide, the word ‘confidence’ is not used to modify the word ‘interval’ when referring to the interval defined by *U* ; and the term ‘confidence level’ is not used in connection with that interval but rather the term ‘level of confidence’. More specifically, *U* is interpreted as defining an interval about the measurement result that encompasses a large fraction *p* of the probability distribution characterized by that result and its combined standard uncertainty, and *p* is the coverage probability or level of confidence of the interval.” — GUM 6.2.2

In its clause 6.1.2, the GUM states that the coverage interval is “expected to encompass a large fraction of the distribution of values that could reasonably be attributed to the measurand.” This may be a more cogent reason not to call it a confidence interval than the reason quoted above. In fact, an interval of this nature would, in normal statistical practice, be more like a credible interval, or a tolerance interval, as will be explained below, in Sec. 3, not a confidence interval.

A conventional confidence interval is designed to cover the true value of a parameter of a probability distribution, or the true value of some known function of the parameters of a probability distribution. In the GUM framework, *y* is not portrayed as such parameter: it is just a function of *n* random variables, hence a random variable, or the realized value of a random variable (our informal notation does not distinguish one from the other).

The GUM’s approach to the evaluation and expression of measurement uncertainty is based on the concept that estimates of measurands are functions of values of input quantities that have been measured previously, or that are measured in the course of the experiment designed to measure the quantity of interest.

The GUM formulates this approach: (1) use a measurement function *f* whose arguments are the input quantities, and whose value is an estimate of the measurand: *y* = *f* (*x*_1_, *. . ., x_n_*); and (2) model the quantities involved as random variables, whose probability distributions describe the uncertainty surrounding their true values.

Since the GUM focuses on scalar measurands, we will do the same here. The function *f*, however, has a special property that the functions usually considered in mathematics do not have: it operates not only on the numerical values of its inputs, but it also preserves their measurement units and transfers them correctly to the output.

For example, consider the measurement model for airspeed using a Pitot tube [4], $v=\sqrt{2\Delta /\rho }$, where Δ (expressed in Pa, i.e., N*/*m^2^) denotes the difference between total and static air pressures, and *ρ* (expressed in kg*/*m^3^) denotes the mass density of air. The measurement function produces a value of the velocity expressed in m*/*s because it is an algebraic function of its arguments and the units are treated as if they were names of mathematical variables.

Some measurement functions are not algebraic, but transcendental. For example, when using the Arrhenius equation [5, Sec. 17D.1] to measure the rate *k* = *A* exp{−*E_α_ /*(*RT*)}, at a particular temperature *T*, of a first-order reaction whose activation energy is *E_α_*, where *R* denotes the gas constant. In this case, the argument of the exponential is unitless, and *k* has the same units, *s*^−1^, as the frequency factor *A*.

This contribution discusses and elucidates the meaning of *coverage interval* as considered in the GUM, and offers a novel interpretation of the meaning of these intervals when they are produced according to the GUM Supplement 1 (GUM-S1) [6].

Section 2 reviews the several different characterizations that the GUM offers for the concept of coverage interval. Section 3 describes and illustrates the principal types of probabilistic intervals that are recognized and widely used in the practice of statistics, and compares them with the coverage intervals proposed by the GUM and by the GUM-S1.

Section 4 offers a novel interpretation for the intervals built following the GUM-S1, which explains much of the criticism leveled against intervals derived from Monte Carlo samples. Section 5 recapitulates the main findings and presents recommendations for how to use and interpret coverage intervals.

## 2 Coverage Intervals

The GUM calculates coverage intervals in three steps:

(1) Compute *y* and evaluate *u*(*y*) using the approximation either in Equation (10) or in Equation (13) in the GUM, depending on whether the input quantities are uncorrelated or correlated.
(2) Assign a probability distribution to *y*: either a Gaussian (or normal) distribution when the uncertainties of the input quantities are all based on infinitely many degrees of freedom, or a rescaled and shifted Student’s *t* distribution in other cases, whose number of effective degrees of freedom is computed using the Welch-Satterthwaite formula (GUM G.4.1).
(3) Calculate a *coverage factor k* such that *y* ± *ku*(*y*) achieves the required coverage probability.

The GUM justifies step (2) based on a linear approximation to the measurement function, assumed to be valid in a suitably small neighborhood of the point, (*ξ*_1_, *. . ., ξ_n_*), whose coordinates are the true values of (*x*_1_, *. . ., x_n_*). If *f* is differentiable, i.e., sufficiently smooth, and *η* = *f* (*ξ*_1_, *. . ., ξ_n_*) denotes the true value of *y*, then we have *f* (*y*) ≈ *η*+ *α*_1_(*x*_1_ − *ξ*_1_)+ · · · + *α_n_*(*x_n_* − *ξ_n_*), where {*α_j_*} denote the values that the first-order partial derivatives of *f* take at (*ξ*_1_, *. . ., ξ_n_*).

This linearization is the basis for the approximations for *u*(*y*) in Equations (10) and (13) of the GUM, and also for invoking the Central Limit Theorem (GUM, Annex G.2). However, considering the example above, for the velocity of air measured using a Pitot tube, and so many others like it, where the number of summands in the linear approximation for *y* is very small, it becomes clear that invoking the Central Limit Theorem (which describes how the distribution of *y* evolves as the number of input quantities becomes very large), is mere wishful thinking.

The more relevant result is the so-called Berry-Esseen bound [7, Sec. XVI.5], which characterizes how close to Gaussian the distribution of a sum of random variables should be, for small or large numbers of summands. However, this result involves the third moments of the input quantities, which usually are neither available nor required because the techniques for uncertainty analysis described in the GUM involve only the first two moments of the random variables used to model the input quantities.

The GUM also effectively assumes that *u*^2^(*y*) is approximately like a multiple of a chi-squared random variable with *ν* degrees of freedom that is independent of *y*. If both this and the foregoing approximation are tenable, then *y* ± *ku*(*y*), with *k* a suitable percentile of Student’s *t* distribution with *ν* degrees of freedom, is an approximate interval covering the specified amount of the probability distribution conveying uncertainty about the true value of *y*. Annex G.4 in the GUM explains how *ν* can be computed, which involves additional assumptions and approximations.

The Monte Carlo method for uncertainty propagation described in the GUM-S1 [6] provides an alternative to the three-step above, requiring neither the preliminary evaluation of *u*(*y*), nor the determination of the factor *k*. Instead, coverage intervals are derived directly from a large sample drawn from the probability distribution of the output quantity.

Hall [8], Willink [9], and Stant *et al.* [10], among others, have criticized particular aspects of the Monte Carlo method. Possolo *et al.* [11] have explained that some of this criticism is deserved not by the Monte Carlo method itself, but by how the GUM-S1 suggests input quantities should be modeled, or how the Monte Carlo sample should be reduced. Possolo and Iyer [4] also have pointed out anomalies that may arise in the use of the Monte Carlo method in practice.

This overview should suffice to suggest that, within the framework of the GUM, the validity of Student’s *t* intervals requires that multiple assumptions be satisfied, none of which is easily verifiable. The Monte Carlo method of the GUM-S1, being able to provide an arbitrarily large sample drawn from the probability distribution of *y*, involves no such set of assumptions and coordinated approximations, yet it requires supplementary criteria whereby this large sample may be reduced to a coverage interval that is ft for purpose.

Therefore, while the GUM-S1 circumvents the demanding assumptions and approximations required to produce a coverage interval within the framework of the GUM, it still leaves unanswered the same question that the GUM falls short of answering: what is the nature of a coverage interval, and what relation does it bear to its cousins that are used routinely in statistical practice, where the term “coverage interval” is not used?

The concept of *coverage interval* is the elephant in the room where the GUM entertains her guests. There is an obvious nervousness in the air about it, because the GUM attempts to explain what it is, and what it is not, on multiple occasions: in Sec. 2.3.5, where it states that the purpose of the expanded uncertainty is to define such an interval; in Sec. 3.3.7, where it suggests that coverage intervals serve “to meet the needs of some industrial and commercial applications, as well as requirements in the areas of health and safety”; and in Sec. 6.2.2, where it emphasizes that a coverage interval is not a confidence interval because such characterization would be “applicable to the interval defined by *U* when certain conditions are met, including that all components of uncertainty that contribute to *u*_c_(*y*) be obtained from Type A evaluations.”

A little later, in Sec. C.2.30, the GUM qualifies the term with the adjective “statistical,” and defines *statistical coverage interval* as “an interval for which it can be stated with a given level of confidence that it contains at least a specified proportion of the population,” yet without explaining which population it refers to. A revealing note adds that it is also called a “statistical tolerance interval,” only to admonish that this term should not be used because it may cause confusion with “tolerance interval,” for whose definition the GUM refers the reader to ISO 3534 [12].

Possolo and Iyer [4, Sec. IV.D.3] provide a concise review of the concepts of *confidence interval*, *credible interval*, *prediction interval*, and *tolerance interval*, and compare instances of them for a specific data set. A (Bayesian) predictive interval may be regarded as a particular kind of prediction interval. Meeker *et al.* [13] discuss at great length statistical intervals that may generally be called *probability intervals*. Some classical statistical intervals as well as their relationship to metrology are reviewed in Ref. [14]. In the next section we begin by reviewing the principal types of probabilistic intervals, pointing out their differences and comparing them with the ways in which the GUM and the GUM-S1 use the term “coverage interval.”

Similarly to how coverage intervals seem to share traits with different types of commonly recognized probabilistic intervals [13], the concept of limit of detection, which is of great importance in analytical chemistry and in measurements of radionuclides, has also been redefined repeatedly and variously in terms of these different types of intervals [15].

## 3 Probabilistic Intervals

We will illustrate the principal types of probabilistic intervals using the following, recent X-ray fluorescence (XRF) determinations of the mass fraction of iron in National Institute of Standards and Technology (NIST) Standard Reference Material™ (SRM) 690, an iron ore powder packaged in 100 g units. This reference material became available in 1978, having been originally measured by five different laboratories, including NIST, using classical and instrumental methods.

Determinations made in duplicate of eight different bottles yielded the following sixteen values for the mass fraction of iron, expressed as percentages (meaning cg*/*g): 67.43, 66.97, 67.65, 66.84, 67.05, 66.57, 67.16, 68.3, 67.01, 67.07, 67.23, 66.51, 66.46, 67.54, 67.09, 66.77.

A conventional analysis of variance of these XRF determinations revealed that the bottle effects are statistically insignificant, and a linear, Gaussian, mixed effects model [16] with bottle as a random effect, fitted to these data by the method of restricted maximum likelihood (REML), estimated the standard deviation of the variance component attributed to between-bottle differences to be 0 cg*/*g and the within-bottle standard deviation to be 0.5 cg*/*g.

This reference material is sufficiently homogeneous for its intended purpose, hence warrants a single value assigned to all its units even though the mass fraction may vary from unit to unit within the margin of uncertainty that surrounds the assigned value. Both the assigned value and the associated standard uncertainty that are listed in the corresponding certificate are based on these XRF determinations and on measurements made by other methods.

We begin by reviewing (sampling theoretic) confidence and (Bayesian) credible intervals for parameters (either the mean or the median) of the probability distribution that the data above originate from. All of them are probabilistic intervals: this means that they have a specified probability of including the true value of the quantity of interest, yet none offers any guarantee that it actually does so.

Confidence intervals have traditionally been built by finding a function of the data and of the parameter of interest whose probability distribution does not depend on this parameter — a so-called *pivot*.

The most famous pivot, for the mean *ω* of a Gaussian distribution whose standard deviation *σ* also is unknown, based on a sample of replicated determinations, *w*_1_, *. . ., w_m_*, is ( $\bar{w}$ − *ω*)*/*$\left(s/\sqrt{m}\right)$), where $\bar{w}$ denotes the average of the determinations, and *s* denotes their sample standard deviation. This pivot has a Student’s *t* distribution with *m* − 1 degrees of freedom, which is independent of both the true mean *ω* and the true standard deviation *σ* [17].

Pivots also play a crucial role when building intervals based on the fiducial approach to statistical inference [18], but we will not discuss these here. Since pivots are not always easy to find, in Sec. 3.1 we also present a general purpose method for producing confidence intervals based on the likelihood function.

A (Bayesian) credible interval for a parameter of a probability distribution is an interval of possible values of the parameter to which the posterior distribution of this parameter assigns a specified probability.

For example, suppose that the observations are a sample from a lognormal distribution, and that one is interested in building a credible interval for the mean of this distribution. The observations, the version of the likelihood function where that mean appears as a parameter, and a prior distribution for the mean together determine the posterior distribution for the mean. Any interval to which this distribution assigns probability 0 *< γ <* 1 is a 100*γ* % credible interval for the mean, conveying the belief that the true mean lies in it with 100*γ* % probability.

Now suppose instead that one is interested in building a credible interval for the median of the lognormal distribution. The observations, the version of the likelihood function where that median appears as a parameter, and a prior distribution for the median, together determine the posterior distribution for the median, whence a credible interval for the median will be built.

If the criterion to build these intervals is that they should be highest posterior density intervals, then the credible intervals for the mean and for the median will be different. This is the reason and sense in which below we often say that the true value of the parameter of interest is the *target* of the credible interval built for it.

Prediction and predictive intervals, considered in Sec. 3.2, characterize the uncertainty surrounding an estimate of the mass fraction of iron in an individual unit of SRM 690 that is sent to a customer. These intervals are wider than their counterparts for the mean of all the units, for the same level of confidence. Prediction intervals can generally be calculated by applying a minor modification to corresponding confidence intervals, and credible intervals can easily be derived from a large sample drawn from the posterior probability distribution of the parameters of the probability distribution of the replicates.

Tolerance intervals (Sec. 3.3) appear to be the closest in concept to the meaning that the GUM ascribes to coverage intervals: they are intended to cover a specified fraction (called *content*) of the unit of probability that a probability distribution allocates to its support, and to do so with some specified confidence. However, they differ obviously from coverage intervals in that their specification involves both a content and a confidence level, while coverage intervals are determined by their coverage probability only.

In the final subsection (Sec. 3.4), we compare the different types and modalities of intervals both numerically and graphically, all derived from the same set of replicate determinations of the mass fraction of iron in NIST SRM 690, but with different modeling assumptions.

### 3.1 Confidence and Credible Intervals

Classical (or sampling-theoretic) *confidence intervals*, and their Bayesian counterparts, *credible intervals*, aim to include the true mean (or the true median, or any other particular characteristic) of the probability distribution of the mass fraction of iron in all units of the material, with some specified probability.

Even when their endpoints are identical, confidence and credible intervals are interpreted differently: the former from a classical viewpoint, the latter from a Bayesian viewpoint.

The classical viewpoint interprets the confidence as the probability that an interval built from a random sample drawn from the distribution that describes the variability of the observations, will straddle the true value of the parameter of interest. That is, from this viewpoint, confidence characterizes the (frequentist, or long-run) performance of the procedure that is used to compute such intervals and does not offer any guarantees about the specific interval derived from the single sample in hand.

Hoekstra *et al.* [19] and Morey *et al.* [20] have shown that the classical interpretation is very often misunderstood, and a plethora of consequential counter-examples challenge the very logic of the classical interpretation [21–23].

The Bayesian viewpoint interprets the confidence as the posterior probability of the target being inside the actual interval derived from the sample in hand, thus remaining unconcerned with how the interval-building procedure performs for samples that have not been drawn (*cf.* [24, Page 385]).

**Fig. 1:**
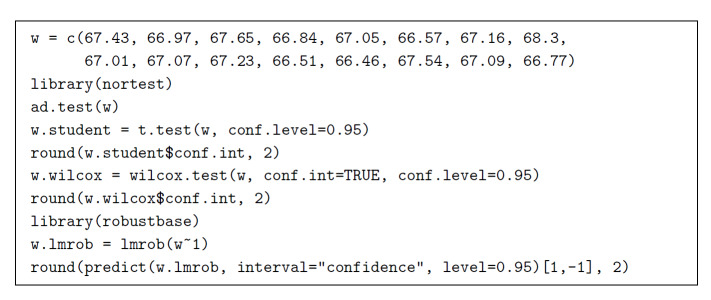
R code to build three confidence intervals for the mean mass fraction of iron in NIST SRM 690, based on 16 replicated determinations. The first is the classical Student’s *t* interval, which can be computed with as few as two numerically distinct replicates. The second is non-parametric and is obtained by inverting Wilcoxon’s signed rank test, requiring at least five numerically distinct replicates. The third is based on robust regression, and can derive a 95% confidence interval from as few as two numerically distinct replicates. The R code to compute the Anderson-Darling test of Gaussian shape is also provided.

The 16 determinations of the mass fraction of iron in NIST SRM 690 have average *$\bar{w}$*= 67.10 cg*/*g and sample standard deviation *s* = 0.47 cg*/*g. The 95% coverage interval according to the GUM Annex G is *$\bar{w}$* ± 2.131*s/$\sqrt{m}$*, where *m* = 16 is the number of replicates and 2.131 is the 97.5th percentile of the Student’s *t* distribution with *m* − 1 = 15 degrees of freedom, hence ranges from 66.85 cg*/*g to 67.35 cg*/*g. This is the same as the conventional Student’s *t* confidence interval for the true mean mass fraction because the uncertainty associated with *$\bar{w}$* is the result of a Type A evaluation. Figure 1 lists the corresponding R code [25].

This coverage interval rests on the assumption that the set of replicate determinations is like a sample drawn from a Gaussian distribution. The Anderson-Darling [26] test of Gaussian shape, applied to the 16 determinations listed above, yields a *p*-value of 0.41, which does not challenge this assumption.

When the assumption of Gaussian shape is questionable, confidence intervals may still be built that are based on less demanding assumptions. One of these requires only that the replicated observations be a sample drawn from a symmetric distribution: it ranges from 66.83 cg*/*g to 67.33 cg*/*g, and was obtained by inversion of Wilcoxon’s signed rank test [27, Sec. 3.2], using the R code listed in Fig. 1. Intervals of this kind are called *non-parametric* because their construction does not involve assumptions about the shape of the probability distribution the sample comes from.

R function lmrob, also used in the code listed in Fig. 1, implements an MM-estimator and produces non-parametric intervals that allow for up to 50% of the data to be moved arbitrarily far away from the other 50% without the estimate deviating from its original value by more than a bounded amount. This MM-estimator is highly efficient in the sense that its variance remains almost as small as it would be if the distribution were actually Gaussian [28, 29]. The term “MM,” which was introduced by V. Yohai [28], alludes to the involvement of two estimates (one of scale, the other of location) that are of maximum likelihood type (even if non-parametric). The procedure offers high breakdown (that is, resistance to a high proportion of abnormally deviant observations) and high efficiency.

The profile likelihood (PL) confidence interval is the result of a model-based, widely applicable procedure that does not involve a pivot. The idea is to build an interval that comprises values of the parameter of interest in the neighborhood of the value that maximizes the likelihood function. Since the model may include other parameters (called *nuisance parameters* in this context) besides the parameter of interest, one defines that neighborhood using the version of the likelihood function where for each value of the parameter of interest, the values of the nuisance parameters are chosen so as to maximize the likelihood function.

In this case, the model is Gaussian, whose likelihood is a function of two parameters, *L***_w_**(*ω, σ*), where the subscript **w** denotes the vector of 16 replicated determinations of the mass fraction of iron in NIST SRM 690, which are fixed, and *ω* and *σ* denote the mean and standard deviation of the Gaussian distribution the {*w_i_*} originate from.

If *$\hat{\omega }$* and *$\hat{\sigma }$* denote the maximum likelihood estimates, then the PL interval is the set of values of *ω* for which max*_σ_ L***_w_**(*ω, σ*)*/L***_w_**(*$\hat{w,}$$\hat{\sigma }$*) ⩾exp(−*χ*_95%_*_,_*_1_*/*2), where *χ*_95%_*_,_*_1_ denotes the 95th percentile of the chi-squared distribution with 1 degree of freedom [13, Sec. 12.5.2].

**Fig. 2:**
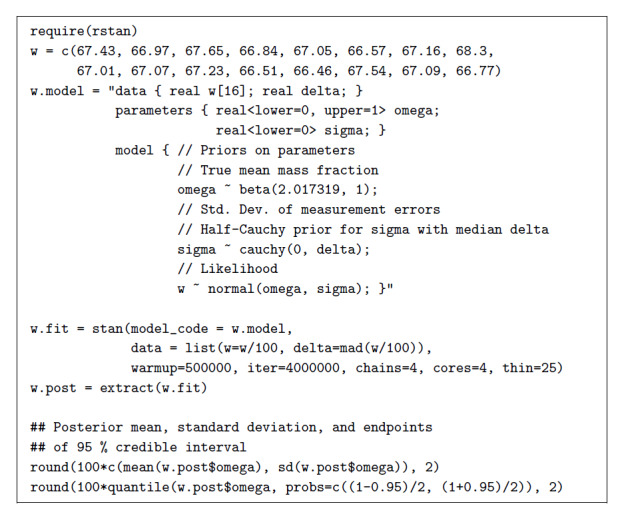
Bayesian model for the determinations of mass fraction of iron in NIST SRM 690, formulated and fitted to the data via Markov chain Monte Carlo sampling [32] using facilities of the Stan language for Bayesian statistical modeling [33], of R package rstan [34]. Note that in the line where function stan is invoked, the value assigned to the data is *w/*100, thus expressing the percentages as proportions consistently with omega having a prior beta distribution, hence lying between 0 and 1.

Figure 2 lists a combination of R and Stan [30] codes that yield a (Bayesian) predictive interval for the same data, ranging from 66.86 cg*/*g to 67.35 cg*/*g, and conforming with the intuitive interpretation that the true value of the measurand is believed to lie within the interval with 95% probability.

The prior distribution assigned to *ω* was a beta distribution with mean 0.66858 g*/*g, where the change in units serves to express the magnitude as a number between 0 and 1, which is the support of the beta distribution. This beta distribution also has the maximum variance possible subject to the constraint that both shape parameters should be no smaller than 1 (to ensure a plausible shape of the overall distribution). The value chosen for the prior mean is the average of five determinations of the same mass fraction that were made by five different laboratories when the material first became available, about 40 years prior to the recent XRF determinations.

The prior distribution assigned to *σ* was half-Cauchy, following Gelman’s recommendation [31], with median set equal to the median of the absolute deviations of the replicated determinations from their median, rescaled to be a consistent estimate of the standard deviation for Gaussian samples, as implemented in R function mad. We also assumed that *ω* and *σ* are independent *a priori*. Both these priors are rather uninformative, yet both are proper: that is, they assign probability 1 to their respective supports.

### 3.2 Prediction and Predictive Intervals

Prediction and predictive intervals are probabilistic statements about the mass fraction of iron in the unit that will be randomly pulled from the shelf and shipped to a customer. This question is relevant in practice because customers only care about the particular units that they receive, after all.

A *prediction interval*, which typically will be appreciably wider than the confidence interval for the mean mass fraction over all the units, is the classical answer to that question. The Bayesian counterpart is called a *predictive* interval, and it is derived from the conditional distribution of a “future” observation given the observations one has already made. A “future” observation refers to the value of the mass fraction of iron that may be measured in an individual unit of the material using an analytical method whose performance is comparable to the methods used for certification.

A slight modification of the GUM’s coverage interval, yields the GUM-like prediction interval with 95% coverage probability: $\bar{w}\pm 2.131s\sqrt{1+1/m}$[13], which ranges from 66.07 cg*/*g to 68.14 cg*/*g. Since the GUM’s coverage interval in this case is the same as the Student’s *t* confidence interval for the mean, this prediction interval rests on the same assumptions.

**Fig. 3:**
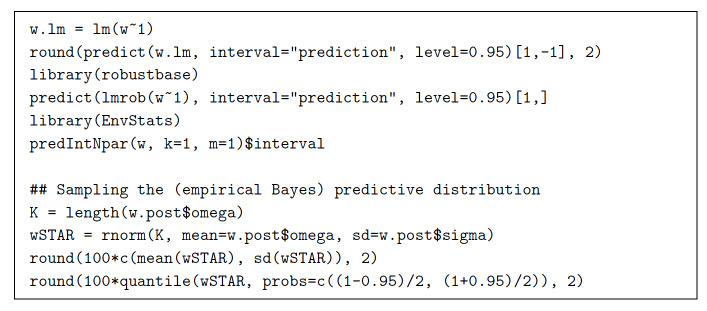
Calculation of three different 95% prediction intervals (first six lines), and calculation of a 95% predictive interval (last four lines) for the true mass fraction of iron in an individual unit of the material, where the latter uses the output of the code in Fig. 2. The actual confidence of the interval produced by R function predIntNpar is only 0.8824. Section 4 explains why this happens to be 15*/*17.

The same R function, lmrob, that was used in the R code listed in Fig. 1, is used again here as listed in Fig. 3, to produce a prediction interval based on a robust statistical procedure, that is, dispensing with the assumption that the observations are a sample from a Gaussian distribution.

R function predIntNpar, defined in package EnvStats [35], produces yet another non-parametric interval, whose endpoints are suitably selected percentiles of the data [13], which is depicted in Fig. 3, where it is labeled NP^∗^. The actual confidence of this interval is only 0.8824. Section 4 explains why this happens to be 15*/*17.

The corresponding (empirical Bayes) predictive interval is derived from the predictive distribution for a “future” observation *w*^∗^, whose probability density is *q*(*w*^∗^|*w*_1_, *. . ., w_m_*) given by

*$\int_{0}^{1}\int_{0}^{+\infty }$ p*(w*|w_1_,…,w_m_,ω,σ|)*q(ω,σ|w_1_,…w_m_)dσdω*

$=\int_{0}^{1}\int_{0}^{+\infty }\frac{p(w_{1},...,w_{m},w*|\omega ,\sigma )}{p(w_{1},...,w_{m}|\omega ,\sigma )}$q(ω,σ|w_1_,…,*w_m_)dσ,dω*

$=\int_{0}^{1}\int_{0}^{+\infty }$ p(w*|ω,σ)q(ω,σ|w_1_,…,w_m_)dσdω,

where *q* is the posterior density of *ω* and *σ* given the data, and *p* is the probability density of the data (and of the “future” observation *w*^∗^) given *ω* and *σ* . The last line follows from the middle line because *w*^∗^ and the {*w_i_*} are mutually independent given *ω* and *σ* .

Instead of computing these integrals, one can sample the predictive distribution by making draws from a mixture of likelihoods with the posterior distribution of *ω* and *σ* as the mixing distribution, as specified in the last four lines of the R code in Fig. 3. This produces a sample whose 2.5th and 97.5th percentiles are the endpoints of the 95% predictive interval sought: (66.08 cg*/*g, 68.13 cg*/*g).

### 3.3 Tolerance Intervals

A *tolerance interval* seeks to cover the values of the mass fraction of iron in a specified proportion of the units, with a specified probability: for example, an interval that will include the values of the mass fraction of iron in 90% of the units, with 95% probability. That proportion (90%) is the *content* of the tolerance interval, and this probability (95%) is its *confidence*.

Tolerance intervals with a particular confidence may be wider or narrower than confidence or credible intervals with the same confidence, depending on the specified content. Hamada *et al.* [36] explain the relation between (Bayesian) predictive intervals and tolerance intervals.

**Fig. 4:**
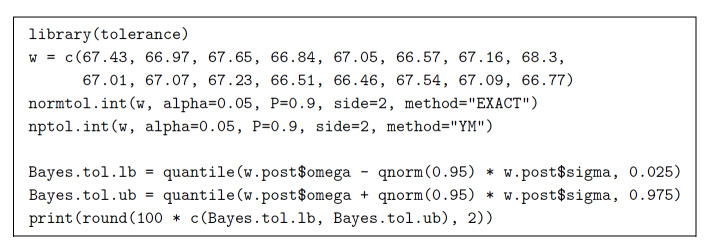
R code to compute three kinds of tolerance intervals: normtol.int and the Bayesian interval require that the replicated determinations of the mass fraction of iron in NIST SRM 690 must be a sample from a Gaussian distribution, while nptol.int involves no such assumption.

Similarly to confidence and prediction intervals, tolerance intervals may be built either making specific assumptions about the probability distribution that the data originate from, or non-parametrically. Figure 4 provides R code for a classical tolerance interval and a Bayesian tolerance interval that assume the data originate from a Gaussian distribution as well as a classical non-parametric tolerance interval. Both classical intervals use functions defined in the package tolerance [37]. The Bayesian interval uses the Stan output from Fig. 2 and the prescription in Sec. 2 of Ref. [36].

### 3.4 Comparing Probabilistic Intervals

Table 1 lists estimates, standard uncertainties, and probabilistic intervals (confidence, credible, prediction, predictive, and tolerance) produced by different statistical procedures, which make different assumptions about the data, or use different models for the same data, and express knowledge either of the reference material as a whole, or of individual units.

**Fig. 5:**
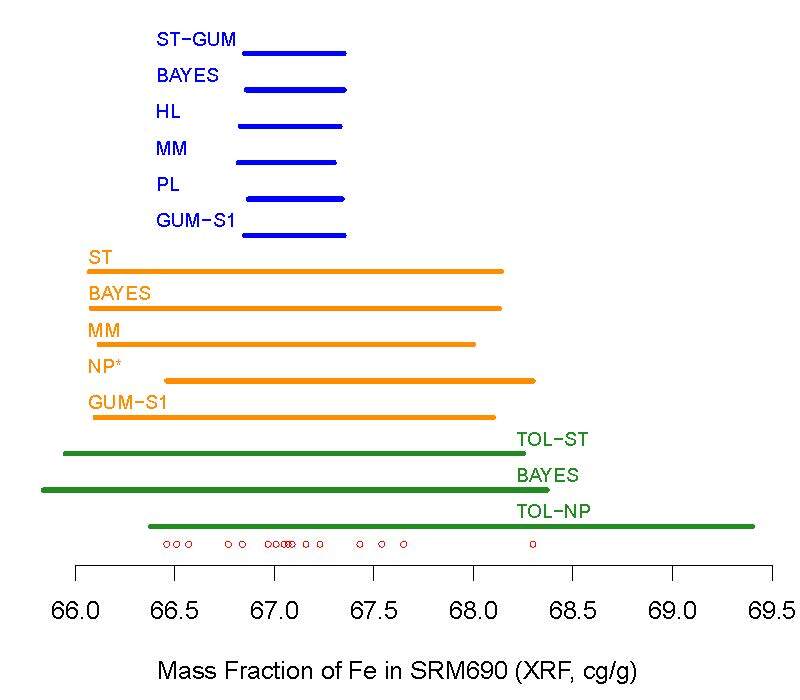
Graphical comparison of the probabilistic intervals listed in Table 1. The different colors indicate their targets: average mass fraction of iron across all units of NIST SRM 690 (blue); mass fraction of iron in a particular unit of the material (orange); mass fraction in 90% of the units (green). The labels are as explained in the caption of Table 1. All these intervals have 95% confidence, except the prediction interval NP^∗^, whose actual confidence, 0.8824, is discussed in Sec. 4. The red, small open circles immediately above the horizontal axis indicate the observations.

The entries are classified according to whether the interval aims to capture the average mass fraction of iron over all the units of NIST SRM 690, or the mass fraction of iron in the single, particular unit that a customer has received. The intervals produced using methods based on the Monte Carlo method of the GUM-S1 are discussed below. Figure 5 depicts all of these intervals.

**Table 1:** **TOP:** Estimates and standard uncertainties (when applicable), and probabilistic intervals for the mean mass fraction of iron in NIST SRM 690 across all units. ST (GUM): conventional Student’s *t*. BAYES: Bayes estimate and credible interval corresponding to the model defined in Fig. 2. HL: Hodges-Lehmann and inversion of Wilcoxon’s signed rank test. MM: MM-estimator implemented in R function lmrob. PL: Profile likelihood (Gaussian model). GUM-S1: same as ST (GUM). **BOTTOM:** Same summaries for a single unit, and for a proportion of the units. ST: Student’s *t* prediction. BAYES: Bayesian prediction corresponding to the same model underlying the credible interval. MM: MM-estimator implemented in R function lmrob. NP^∗^: non-parametric prediction. GUM-S1: Prediction. TOL-ST, BAYES, and TOL-NP are tolerance intervals with 90% content (that is, aiming to include 90% of the values of the mass fraction in individual units of the material), where the first is based on Student *t*, the second is Bayesian, and the third is non-parametric. All intervals have 95% confidence, except NP^∗^, whose actual confidence is 0.8824.

Mean mass fraction over all units of NIST SRM 690 METHOD ESTIMATE STD. UNC. PROB. INTERVAL
*Confidence or Credible Intervals*
ST (GUM) 67.10 0.12 (66*.*85*, *67*.*35)*/*(cg*/*g)
BAYES 67.10 0.12 (66*.*86*, *67*.*35) HL 67.06 0.11 (66*.*83*, *67*.*33) MM 67.06 0.11 (66*.*82*, *67*.*30) PL 67.10 0.11 (66*.*87*, *67*.*34)
*GUM-S1 Interval*
67.10 0.13 (66*.*85*, *67*.*35)
Mass fraction in individual unit of NIST SRM 690 METHOD ESTIMATE STD. UNC. PROB. INTERVAL
*Prediction Intervals*
ST 67.10 0.47 (66*.*07*, *68*.*14) BAYES 67.10 0.52 (66*.*08*, *68*.*13)*/*(cg*/*g) MM 67.06 0.11 (66*.*12*, *68*.*00)
*GUM-S1 Interval*
7.10 0.47 (66*.*10*, *68*.*10)
*Tolerance Intervals*
TOL-ST 67.10 (65*.*95*, *68*.*25) BAYES (65*.*84*, *68*.*37) TOL-NP (66*.*38*, *69*.*40)

Section 4 explains why this happens to be 15*/*17. These intervals are depicted in Fig. 5.

The intervals for the mean mass fraction (depicted in blue) are all fairly similar to one another, including the credible interval (BAYES) because the prior distributions used, although proper, are rather uninformative. The prediction intervals (depicted in orange) and the tolerance intervals (depicted in green) also are quite similar to one another within the same type of interval. Both non-parametric intervals, one for prediction (NP^∗^) and the other tolerance (TOL-NP), are perceptibly shifted to the right relative to the others, because they track the largest determination, owing to the fairly small number of determinations.

The endpoints of the GUM-S1 coverage interval are the 2.5th and 97.5th percentiles of a large sample drawn as described in the GUM-S1 (6.4.9.). However, no sampling would have been needed considering that the modeling choices in 6.4.9. of the GUM-S1 imply that the interval should be exactly the same as the Student’s *t* interval, ST (GUM).

The endpoints of the non-parametric prediction interval produced by predIntNpar are the extremes (smallest and largest) of the 16 determinations of the mass fraction of iron. The same function also outputs the actual confidence that this interval achieves, 0.8824, which happens to be 15*/*17. In Sec. 4 we will explain how this fraction arises.

The endpoints of the GUM-S1 prediction interval are the 2.5th and 97.5th percentiles of a sample drawn from a rescaled and shifted Student’s *t* distribution with 16 degrees of freedom. The scale factor is the sample standard deviation, *s* = 0.47 cg*/*g, and the shift centers the distribution at the sample average, 67.10 cg*/*g.

With all of these options for intervals that express uncertainty, where does the GUM stand? The GUM leans toward an interpretation of coverage intervals that conveys the state of incomplete knowledge about the measurand. In this interpretation the coverage probability quantifies incompleteness of knowledge, thus suggesting that coverage intervals are closest in spirit to credible intervals. However, the GUM does not describe Bayesian procedures, nor does it present a single instance of a Bayesian credible interval.

The GUM-S1 does produce a Bayesian credible interval for the mean mass fraction following its 6.4.9, but at the cost of making assumptions that are unrealistic and defy common sense. However, these efforts are inconsequential because the interval it produces as a result, is none other than the conventional, Student *t* interval we have had all along and that the GUM promotes. Its predictive counterpart can also, in this particular case, and just as easily, be computed in closed form, without any Monte Carlo sampling.

In this particular situation, involving an average of a sample from a Gaussian distribution, the GUM-S1 leverages the magic surrounding the pivot ($\bar{w}$− *ω*)*/$\left(s/\sqrt{m}\right)$*, where *ω* represents the true mean and *m* is the number of observations. This pivot’s probability distribution is known in closed form (Student’s *t*), and does not involve the unknown standard deviation *σ* of the underlying Gaussian distribution that the observations have been drawn from.

All of the parametric intervals from Table 1, which are depicted in Fig. 5, assume that the dispersion of the 16 determinations of the mass fraction of iron in SRM 690 are well described by a Gaussian distribution. It just so happens that a lognormal distribution fts the 16 observations of mass fraction even better than a Gaussian distribution does. Had we used the lognormal model instead, then the GUM-S1 would have had to follow the usual route, and actually draw samples from the distribution of the output quantity. In addition, with the lognormal model the focus on the average would no longer be as natural a choice as it is for the Gaussian model.

Another peculiarity of this example is the fact that we have replicated observations of the quantity of interest itself, which is the mass fraction of iron in this reference material. We can then focus either on the mean value of the distribution the observations come from, or on individual, “future” observations.

In general, however, we do not have replicates of the quantity of interest, which is the output quantity from the measurement model in the GUM, *y* = *f* (*x*_1_, *. . ., x_n_*): instead, we have but a single value of the output quantity. In this more general setting, which we will pursue in the next section, all that the GUM-S1 interval can do is capture a specified proportion of the distribution of the output quantity, rather than aim to capture a particular characteristic of the distribution of *y*, like its mean, which is the target of confidence or credible intervals. In due course, we will conclude that the GUM-S1 interval is a hybrid interval, combining parametric and non-parametric features, and in fact delivering a prediction interval for “future” values of the output quantity.

### 4 Interpreting Monte Carlo Coverage Intervals

The meaning of *coverage interval* is important: first, because the GUM gives it pride of place, and second because Monte Carlo methods of the kinds described in the GUM Supplements 1 and 2 [6, 38] are being used increasingly often to produce such intervals. Every time the *NIST Uncertainty Machine* (https://uncertainty.nist.gov) is invoked to perform uncertainty propagation, it always provides the results of the Monte Carlo method alongside the results obtained using the conventional techniques of the GUM.

Possolo and Iyer [4, Sec. VII.A.4] devised a realistic example involving the Pareto distribution where, in the absence of clairvoyance, it is impossible to produce a non-trivial interval that includes the true mean value of the measurand with specified probability. However, they also open a door toward a better understanding of the meaning of coverage intervals derived from Monte Carlo samples. Since this understanding may be the key to resolving several of the issues that have been raised about intervals produced in this way [8, 39, 40], it is worth exploring the landscape that open door reveals, which we will pursue next.

Consider the measurement model proposed in the GUM once again, which expresses the measurand as a known function of several input quantities and models the uncertainty surrounding the input quantities by assigning probability distributions to these quantities, in effect rendering them as random variables.

In consequence, the output quantity, *y* = *f* (*x*_1_, *. . ., x_n_*), becomes a random variable itself, whose probability distribution is fully determined by *f* and by the joint probability distribution of *x*_1_, *. . ., x_n_*. If the function *f* is suitably smooth, then it is possible to write down a formula for *y*’s probability density in terms of the probability density of the inputs: the so-called *change-of-variable* formula [41].

However, in most instances of application it is impractical to use this formula to carry out computations involving the probability distribution of *y*. The Monte Carlo method circumvents this difficulty by drawing samples from *y*’s distribution without computing this distribution first. It does this by repeatedly making drawings (each of which is an *n*-dimensional vector of values of the *n* input quantities) from the joint probability distribution of the input quantities, and for each such drawing computes a value of the output quantity.

Let *y*_1_, *y*_2_, *. . ., y_K_* denote the results of such a procedure, which are a sample, typically of a large size *K*, from the distribution of the output quantity. Sort these values from smallest to largest, and denote the result *y*_(1)_, *y*_(2)_, *. . ., y*_(_*_K_*_)_. That is, *y*_(1)_ is the smallest of the {*y_k_*}, *y*_(2)_ is the second smallest, and so on, with *y*_(_*_K_*_)_ being the largest. The {*y*_(_*_k_*_)_} are called the *order statistics* of the sample {*y_k_*}.

The differences between successive order statistics, *s_k_* = *y*_(_*_k_*_+1)_ − *y*_(_*_k_*_)_, for *k* = 1, *. . ., K* − 1, are the *spacings*. We will refer to the corresponding sub-intervals (−∞, *y*_(1)_), (*y*_(1)_, *y*_(2)_), . . ., (*y*_(_*_K_*_−1)_, *y*_(_*_K_*_)_), (*y*_(_*_K_*_)_, +∞) as the *slots*. Since we assume that *y* has a continuous distribution, it makes no difference whether the slots are defined as open or semi-closed intervals.

To make these quantities concrete, and as preparation for what will come next, let us consider a toy example where, unbeknownst to us, the output quantity has a Weibull probability distribution with shape 1.5 and scale 1. Best to visualize the features we wish to highlight, we will do something that is never done when the Monte Carlo method is used in practice: we will draw only *K* = 7 values from this Weibull distribution that we assume is the distribution of the output quantity. (In practice, *K* is typically around 1 million.)

The following toy example involves neither classical confidence intervals nor (Bayesian) credible intervals. Its sole purpose is to exploit basic facts about the probabilistic structure of samples drawn from any probability distribution and to produce a simple interpretation of coverage intervals derived from a sample drawn from a probability distribution for the output quantity, *y*, in a conventional measurement model, according to the GUM Supplement 1 [6].

**Fig. 6:**
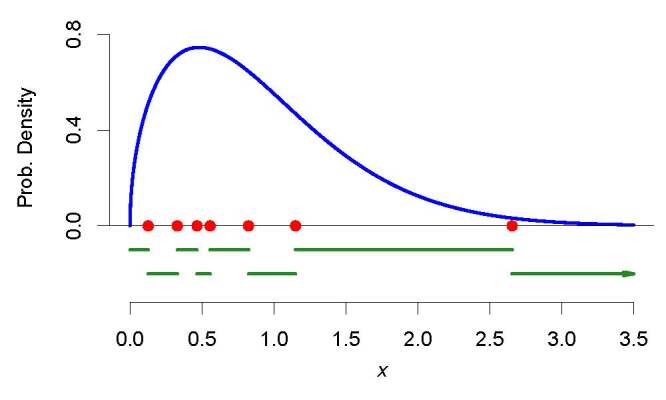
The (blue) curve represents the true probability density of the output quantity, the (red) dots represent a sample of size *K* = 7 drawn from this distribution, and the (dark green) horizontal segments, in alternating lines for better visibility, indicate the slots: their lengths are the spacings. Note that, in this case, because the output quantity is positive, the first slot is an interval of finite length, extending from 0 to the first order statistic. Note that the *K* + 1st slot is a half-line of infinite length (suggested by the arrowhead) starting at the *K*th order statistic, because the output quantity may take arbitrarily large values.

Let us then begin by supposing that our tiny sample drawn from the distribution of *y* comprises these values: 0.1258, 0.8234, 2.6557, 0.5563, 1.1503, 0.3284, and 0.4660. The corresponding order statistics are 0.1258, 0.3284, 0.4660, 0.5563, 0.8234, 1.1503, and 2.6557. The first spacing is 0.3284 − 0.1258 = 0.2026, and the others are 0.1376, 0.0903, 0.2671, 0.3269, and 1.5054, respectively. The order statistics, and the slots they define, are depicted in Fig. 6.

Now, consider this question: what is the probability that the next drawing we will make from the probability distribution of the output quantity, will land in any particular one of these slots? If we knew the actual distribution (we are acting on the pretense that we do not know it), we could answer readily that the probabilities are 0.044, 0.128, 0.101, 0.067, 0.187, 0.183, 0.278, 0.013, for the first, second, etc. slots, respectively. For example, 0.044 = Pr{*y <* 0.1258}, 0.128 = Pr{0.1258 *< y <* 0.3284}, and similarly for the others, except for the last, which is 0.013 = Pr{*y >* 2.6557}. These probabilities can all be computed using the cumulative distribution function of the Weibull distribution with shape 1.5 and scale 1.

The probabilities associated with these slots are obviously different, which, in light of Fig. 6, is not surprising. However, if we reformulate the question ever so slightly, we will get a very surprising answer. The reformulated question is this: what is the probability that the *K* + 1st drawing from the probability distribution of the output quantity will land in any particular one of the slots corresponding to the order statistics of the first *K* drawings?

The original question referred specifically to the drawings we had already made, which happened to have had the values that we listed. The reformulated question refers not to any particular set of *K* = 7 values drawn from the distribution of *y*, but to any and all hypothetical sets of *K* = 7 values that may be drawn from the same distribution. The original question is the kind of question that a Bayesian predictive distribution would answer. The reformulated question is the kind of question that the Monte Carlo method answers.

The surprising answer, which is validated in Appendix A, is that all slots have the same probability of containing the value produced in the next draw, 1*/*(*K* + 1), which in this case is 1*/*(7 + 1) = 0.125. Appendix A (Sec. 6) shows that this can be verified empirically by carrying out a computer experiment, and it can also be proved with great generality.

The foregoing considerations imply that the union of any *M* ⩽ *K* − 1 of the slots {(*y*_(_*_k_*_−1)_, *y*_(_*_k_*_)_)} (which can be chosen so that their union is an interval) is a probability interval for *y* with coverage approximately *M/*(*K* + 1). However, this interval can say nothing about the mean, or about the median, or any other similar attribute of the distribution of *y*. Failure to recognize this fact is the cause of much of the criticism leveled against intervals derived from Monte Carlo samples. Anyone who expects these intervals to deliver more than they are capable of delivering inevitably will be disappointed.

It is worth comparing a coverage interval produced following the GUM-S1 prescription with an alternative probabilistic interval. Since the coverage probability associated with each slot (little interval between successive, ordered sample values) is 0.125, by gluing together the first seven slots we achieve 87.5% coverage. (Note that the very first slot goes from 0 to the smallest sample value.) This will then be the GUM-S1 interval. The interval ranges from 0 to 2.6557 (the largest value in the sample).

**Fig. 7:**
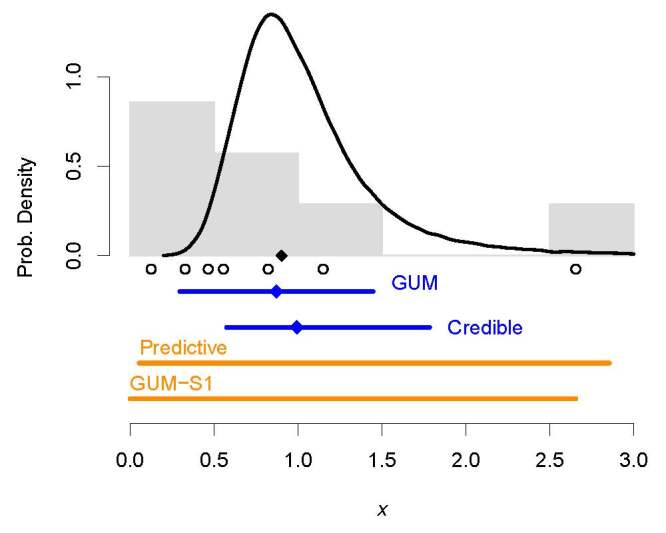
Toy example involving a Monte Carlo sample of size *K* = 7, represented by the open black circles under their probability histogram (gray rectangles). The output quantity is assumed to have a Weibull distribution with true mean 0.9027, indicated by the black, solid diamond. The skewed (black) curve is the probability density of the posterior distribution of the Weibull mean given the sample of size 7 drawn from it. The dark blue horizontal line segments are 87.5% probability intervals: one is the GUM (Student’s *t*) interval; the other is a credible interval. The solid blue diamonds indicate the corresponding estimates of the Weibull mean. The dark orange line segments are 87.5% probability intervals for a single, “future” drawing of the output quantity: one is a predictive interval, the other is an interval built according to the GUM-S1. The latter is the union of seven slots each of probability 1*/*(*K* + 1) = 1*/*8, therefore with coverage probability 7*/*8 = 87.5%.

Figure 7 shows a probability histogram of the *K* = 7 values we sampled from the probability distribution of the output quantity. It also shows an estimate of the posterior probability density of *µ* derived from this sample, and four probability intervals that one might have hoped would capture the true mean, and that, in this case, actually do. The interval labeled GUM is not appropriate here because it assumes that the output quantity has a Gaussian distribution while we know that the output quantity has a Weibull distribution.

In this example, which is more typical of the application of the Monte Carlo method than the example used in Sec. 3, the GUM-S1 interval is nothing like either the conventional GUM interval or the credible interval. The reason is that both these intervals deliberately target the mean of the distribution of *y* for coverage, while the GUM-S1 interval does all that it can do, which is to capture a specified portion of the unit of probability of the distribution of the output quantity.

These facts support the suggestion made near the end of Sec. 3, to the effect that the GUM-S1 interval has a hybrid character, combining parametric and non-parametric traits: (1) the parametric trait involves making draws from a probability distribution determined by the distributions of the inputs, which in most cases are parametric distributions like the Gaussian, rectangular, triangular, *etc.*; (2) the non-parametric trait is the way in which the interval is built, by selecting percentiles of the Monte Carlo sample for its endpoints, which is tantamount to gluing together equal-probability slots between consecutive order statistics.

At this juncture we are ready to explain why the non-parametric prediction interval depicted in Fig. 5, labeled NP^∗^ (orange), has the peculiar probability of 0.8824, which happens to be 15*/*17, instead of the 0.95 that was requested when function predIntNpar was invoked. The reason is that the interval results from gluing the 15 slots together that lie between the smallest and largest value of the sample, each of which has probability 1/17 of containing the “next” draw from the distribution of *y*.

No probabilistic interval is assured to cover any particular characteristic of the probability distribution of the output quantity, be it the mean, the median, or any other. However, intervals like the GUM’s, as well as credible intervals, target specific characteristics by design (in the foregoing example both target the mean of the distribution of the output quantity), and will achieve their nominal coverage provided all the assumptions that validate them are satisfied.

Not so with the GUM-S1 intervals, for the simple reason that their target is elusive: it is the “next” drawing, any value that shall be drawn at random from the probability distribution of the output quantity. The coverage the GUM-S1 intervals achieve, by construction, is of a proportion of the unit of probability of the distribution of the output quantity, not of any particular characteristic of this distribution. Appendix B describes an extreme example that exposes the fickleness of the GUM-S1 intervals.

This section sought to clarify the true nature of the GUM-S1 intervals, attempted to explain the information they provide reliably, and dispelled misplaced hopes that have motivated complaints against their performance. In particular, complaints were addressed that relate to these intervals not achieving their nominal coverage when effective coverage is gauged in terms of their covering specific targets like the mean or the median of the probability distribution of the output quantity.

### 5 Summary and Recommendations

We have reviewed and compared a wide variety of probabilistic intervals with coverage intervals as defined and illustrated in the GUM. The formal construction of coverage intervals in the GUM is the same as the construction of classical confidence intervals, even if the interpretation that the GUM gives of them is more akin to the meaning of Bayesian credible intervals, or of tolerance intervals, as these are defined and used in the practice of statistics.

We have focused in particular on intervals based on samples drawn from the probability distribution of the measurand by application of the Monte Carlo method of the GUM-S1. In some cases, the actual coverage of particular characteristics of the distribution of the output quantity, like the mean, that these intervals achieve is strikingly different from their nominal coverage probability.

We explain this discrepancy by showing that, in general, the GUM-S1 intervals aim to cover a specified proportion of the distribution of the output quantity, and so they are rather different from confidence intervals for a particular parameter of the distribution of this quantity. In some particular cases, for example when the focus is on the average of a Gaussian sample, the GUM-S1 intervals achieve nominal coverage because sampling is from the distribution of the average.

The GUM-S1 intervals aim to capture a “typical” value of the output quantity, with specified probability, not necessarily to capture the mean, or the median, or any other similar attribute of the distribution of this quantity. Realizing this fact should help tune expectations about what these GUM-S1 intervals actually can deliver in practice.

## Figures and Tables

**Fig. 8:**
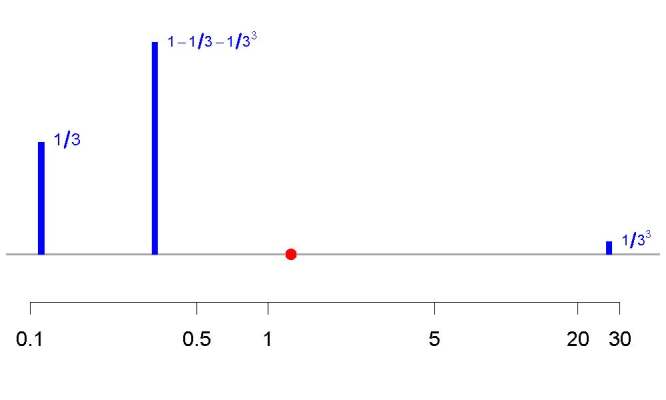
The Nemesis probability distribution for 75% coverage has *ν* = 3. The values to which it assigns positive probability, 1*/*3^2^, 1*/*3, and 3^3^, are located where the vertical, solid (blue) bars stand, and the heights of these bars represent the probabilities assigned to those values. The solid (red) dot indicates the mean of the distribution. Note that the horizontal axis has a logarithmic scale. Since 1*/*3 + 1 − 1*/*3 − 1*/*3^3^ = 26*/*27 is sufficiently greater than 75%, the upper endpoint of the interval will always be the middlemost point, which lies to the left of the mean.

**Fig. 9:**
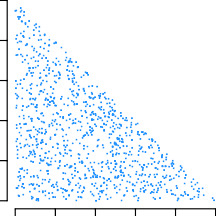

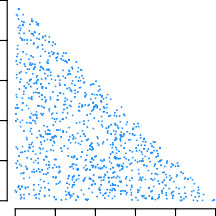

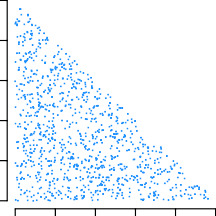

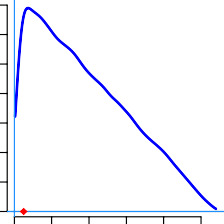
Sample of size 1000 drawn from the Dirichlet(1, 1, 1) prior distribution for (*π*_1_, *π*_2_, *π*_3_) (three leftmost panels), and corresponding prior density for *µ_ν_* (rightmost panel). The red diamond indicates the prior mean of *µ_ν_* .

## 6 Appendix A: Probabilities for Slots Between Order Statistics

In Sec. 4 we provided a surprising answer to a question about the probability with which a “future” observation will land in a particular slot between consecutive order statistics. Here we explain how to verify this answer empirically, and justify it rigorously.

First, select a large integer *N* and choose a value for *K*, which may be the same as we chose above, *K* = 7, or any other. Set the counters *C*_1_, *. . ., C_K_*_+1_ all equal to 0 at the outset. Then repeat the following steps for *n* = 1, *. . ., N*:

(a) Draw a sample of size *K* from the probability distribution of the output quantity;

(b) Compute the order statistics of this sample, which partition the range of *y* into *K* + 1 slots;

(c) Make the “next” drawing, *y_K_*_+1_, from the probability distribution of the output quantity;

(d) Determine the slot where *y_K_*_+1_ landed. If it was slot *j*, then increase the corresponding counter by 1: that is, replace *C_j_* with *C_j_* + 1.

Each counter *C_j_* has a binomial distribution based on *N* trials, and one can then perform a statistical test of whether the corresponding probability of “success” is 1*/*(*K* + 1) — and it will be. Next, we will explain why.

Let *G* denote *y*’s cumulative distribution function. That is, *G*(*s*) is the probability that *y* ⩽ *s* for any possible value *s* that *y* can take. Let *r*_1_, *r*_2_, *. . ., r_K_* denote independent random variables with rectangular (that is, uniform) distributions concentrated on the interval (0, 1).

In these circumstances, *G*^−1^(*r*_(1)_), *G*^−1^(*r*_(2)_), . . ., *G*^−1^(*r*_(_*_K_*_)_) have the same joint distribution as *y*_(1)_, *y*_(2)_, . . ., *y*_(_*_K_*_)_, where *G*^−1^ is the mathematical inverse (not the arithmetic reciprocal) of *G*. Note that *r*_(1)_ ⩽ *r*_(2)_ ⩽ · · · ⩽ *r*_(_*_K_*_)_ are the order statistics corresponding to the {*r_i_*}.

The probability of *y* being in the interval (*y*_(_*_k_*_−1)_, *y*_(_*_k_*_)_) is

$\pi k=\int_{0}^{1}\left\{\int_{0}^{r(k)}\left[G\left(G^{-1}(r_{\mathrm{(k)}}\right)-G\left(G^{-1}\left(r_{\mathrm{(k-1)}}\right)\right)\right]p(r_{\mathrm{(k)}},r_{\mathrm{(k-1)}})dr_{\mathrm{(k-1)}}\right\}dr_{\mathrm{(k)}}),$

where *p*(*r*_(_*_k_*_)_, *r*_(_*_k_*_−1)_) is the joint probability density of (*r*_(_*_k_*_)_, *r*_(_*_k_*_−1)_). Since the (unordered) {*r_k_*} follow a standard uniform distribution, *π_k_* reduces to

$\frac{K!}{(k-2)!(K-k)!}\int_{0}^{1}\left\{\int_{0}^{r(k)}(r_{\mathrm{(k)}}-r_{\mathrm{(k-1)}})r_{\left(k-1\right)}^{k-2}(1-r_{\mathrm{(k)}}K-kdr_{\mathrm{(k-1)}}\right\}dr_{\mathrm{(k)}})=\frac{1}{K+1}.$

## 7 Appendix B: Nemesis

Here we present an example that illustrates, in an artificial but particularly cogent way, how a coverage interval built according to the GUM-S1 may have zero effective coverage frequency for the mean of a distribution, regardless of how high the specified coverage probability may be. Think of it as a game that the metrologist plays against the Greek goddess *Nemesis* (who exacts retribution against pride and arrogance), involving a measurand whose true value is positive.

The metrologist seeks an interval of the form (*A, B*) that, with specified probability 0 *< γ <* 1, will include the true mean of a probability distribution for the measurand, and the metrologist has resolved to employ the Monte Carlo method of the GUM-S1 to build it, by setting *A* and *B* equal to the 100(1 − *γ*)*/*2 and 100(1 + *γ*)*/*2 percentiles of the Monte Carlo sample.

Learning of this desire, and being told the confidence level *γ* the metrologist requires, *Nemesis* then produces a probability distribution that the metrologist can sample at will, using the Monte Carlo method, to determine *A* and *B*. Much to the metrologist’s chagrin, no matter what value the metrologist will have chosen for *γ*, and regardless of how large a sample the Monte Carlo method will draw from the distribution that *Nemesis* concocted, the interval (*A, B*) *never* includes the mean the metrologist seeks to bracket.

The *Nemesis* probability distribution, depicted in Fig. 8 for one particular case, assigns probabilities *π*_1_ = 1*/ν*, *π*_2_ = 1 − 1*/ν* − 1*/ν*^3^, and *π*_3_ = 1*/ν*^3^ to *ξ*_1_ = 1*/ν*^2^, *ξ*_2_ = 1*/ν*, and *ξ*_3_ = *ν*^3^, respectively, where *ν* is the smallest positive integer such that *π*_1_ + *π*_2_ *>* (1 + *γ*)*/*2 and *µ_ν_ > F*^−1^((1 + *γ*)*/*2), with *F_ν_*^−1^ denoting the quantile function (mathematical inverse of the cumulative distribution function) of the *Nemesis* distribution, whose mean is *µ_ν_* = 1 + (*ν*^2^ + 1)(*ν* − 1)*/ν*^4^.

However, it is possible to choose a prior distribution for *µ_ν_* that, together with the likelihood function corresponding to the *Nemesis* distribution, produces credible intervals whose actual, frequentist coverage of the true value of *µ_ν_* is approximately equal to the nominal confidence *γ*.

This is yet another illustration of the fact, already pointed out in the foregoing, that while the GUM-S1 interval is blind to the whereabouts of *µ_ν_*, the credible interval derived from the posterior distribution of *µ_ν_* does have the true value of *µ_ν_* as its target, and does “catch” it under repeated sampling with frequency close to the nominal coverage probability.

Assign a Dirichlet prior distribution to (*π*_1_, *π*_2_, *π*_3_), with parameters (1, 1, 1), which renders (*π*_1_, *π*_2_, *π*_3_) uniformly distributed inside the two-dimensional simplex [42, Chapter 49]. This induces a prior distribution for *µ_ν_* = *ξ*_1_*π*_1_ + *ξ*_2_*π*_2_ + *ξ*_3_*π*_3_ (whose density is depicted in the rightmost panel of Fig. 9).

If *n* denotes the size of a sample drawn from the *Nemesis* distribution, and *N*_1_, *N*_2_, and *N*_3_ are the numbers of observations in this sample that are equal to *ξ*_1_, *ξ*_2_, and *ξ*_3_, respectively, then (*N*_1_, *N*_2_, *N*_3_) has a multinomial distribution, whose conjugate distribution is the Dirichlet distribution [43, Appendix A].

R function MCmultinomdirichlet, which is defined in package MCMCpack [44], may then be used to sample the corresponding posterior distribution of (*π*_1_, *π*_2_, *π*_3_), from which a sample from the posterior distribution of *µ_ν_* can be computed, and a credible interval for *µ_ν_* found that has the specified coverage probability, *γ*.

Provided the sample size, *n*, is sufficiently large (say, *n* = 53 when *ν* = 3) so that all three values in the support of the *Nemesis* distribution are expected to occur at least once in the sample, such credible intervals, under repeated sampling (from the *Nemesis* distribution), cover the true value of *µ_ν_* with frequency close to the nominal coverage probability *γ*.
